# Trends of Multimorbidity Patterns over 16 Years in Older Taiwanese People and Their Relationship to Mortality

**DOI:** 10.3390/ijerph19063317

**Published:** 2022-03-11

**Authors:** Hsin-En Ho, Chih-Jung Yeh, James Cheng-Chung Wei, Wei-Min Chu, Meng-Chih Lee

**Affiliations:** 1 Institute of Medicine, Chung Shan Medical University, Taichung 40201, Taiwan; hsianen@gmail.com (H.-E.H.); jccwei@gmail.com (J.C.-C.W.); 2 Department of Family Medicine, Taichung Armed Forces General Hospital, Taichung 41152, Taiwan; 3 School of Medicine, National Defense Medical Center, Taipei 11490, Taiwan; 4 School of Public Health, Chung-Shan Medical University, Taichung 40201, Taiwan; alexyeh@csmu.edu.tw; 5 Department of Allergy, Immunology & Rheumatology, Chung Shan Medical University Hospital, Taichung 40201, Taiwan; 6 Graduate Institute of Integrated Medicine, China Medical University, Taichung 40402, Taiwan; 7 Department of Family Medicine, Taichung Veterans General Hospital, Taichung 40705, Taiwan; 8 School of Medicine, National Yang Ming Chiao Tung University, Taipei 11221, Taiwan; 9 Department of Post-Baccalaureate Medicine, College of Medicine, National Chung Hsing University, Taichung 40227, Taiwan; 10 School of Medicine, Chung Shan Medical University, Taichung 40201, Taiwan; 11 Institute of Health Policy and Management, National Taiwan University, Taipei 10617, Taiwan; 12 Department of Family Medicine, Taichung Hospital, Ministry of Health and Welfare, Taichung 40343, Taiwan; 13 Institute of Population Sciences, National Health Research Institutes, Miaoli 35053, Taiwan; 14 College of Management, Chaoyang University of Technology, Taichung 41349, Taiwan; 15 Study Group of Integrated Health and Social Care Project, Ministry of Health and Welfare, Taipei 11558, Taiwan

**Keywords:** multimorbidity, trend, elderly, mortality, latent class analysis, chronic diseases

## Abstract

Understanding multimorbidity patterns is important in finding a common etiology and developing prevention strategies. Our aim was to identify the multimorbidity patterns of Taiwanese people aged over 50 years and to explore their relationship with health outcomes. This longitudinal cohort study used data from the Taiwan Longitudinal Study on Aging. The data were obtained from wave 3, and the multimorbidity patterns in 1996, 1999, 2003, 2007, and 2011 were analyzed separately by latent class analysis (LCA). The association between each disease group and mortality was examined using logistic regression. Four disease patterns were identified in 1996, namely, the cardiometabolic (18.57%), arthritis–cataract (15.61%), relatively healthy (58.92%), and multimorbidity (6.9%) groups. These disease groups remained similar in the following years. After adjusting all the confounders, the cardiometabolic group showed the highest risk for mortality (odds ratio: 1.237, 95% confidence interval: 1.040–1.472). This longitudinal study reveals the trend of multimorbidity among older adults in Taiwan for 16 years. Older adults with a cardiometabolic multimorbidity pattern had a dismal outcome. Thus, healthcare professionals should put more emphasis on the prevention and identification of cardiometabolic multimorbidity.

## 1. Introduction

Taiwan has been an aging society since 2018 and is expected to be a super-aged society in 2025 [1], suggesting that one in five citizens will be over 65 years old. As the aging population rapidly grows, public issues, especially medical demands, also grow. Multimorbidity, defined as the coexistence of two or more chronic conditions in a person, is frequently observed on older people, leading to problems of polypharmacy and increased difficulty to provide care [2,3]. Multimorbidity is also associated with worse clinical outcomes, poorer quality of life, and increased medical expenditures [4,5,6].

Multimorbidity has been widely measured by disease numbers or severity [7,8], but rarely by disease patterns. Certain conditions are more likely to cluster than others. They may share causal factors and have similar or the same pathological pathways or networks; among them are cardiovascular diseases and some metabolic diseases [9,10]. Mapping the disease clusters may help us find the possible etiology of multimorbidity, allowing us to better understand which multimorbidity clusters cause the greatest burden and to identify the determinants of the most common clusters of conditions; as a result, the prevention strategy of different clusters of multimorbidity could be developed.

Latent class analysis (LCA) is a statistical procedure used to identify different subgroups within populations who often share similar characteristics [11]. Hence, recent studies have used LCA to determine disease patterns. In the United States, participants aged 50 years and older with different combinations of 13 chronic conditions were surveyed from 2002 to 2014 by the National Health Interview Survey. Five multimorbidity groups were identified, namely, healthy (51.5%), age-associated chronic conditions (33.6%), respiratory conditions (7.3%), cognitively impaired (4.3%), and complex cardiometabolic (3.2%). The cognitively impaired group demonstrated a significantly higher mortality [12]. Moreover, a Korean study discovered three disease patterns: a relatively healthy group (60.4%); a cardiometabolic conditions group (27.8%); and an arthritis, asthma, allergic rhinitis, depression, and thyroid disease group (11.8%) [13].

However, no similar study has been conducted in Taiwan. Considering that Taiwan is one of the most rapidly aging countries worldwide, understanding its multimorbidity pattern over the years will help us understand its process and the impact of aging. This study aimed to identify the disease patterns of Taiwanese people aged over 50 years and to explore their relationship to health outcomes through a population-based longitudinal study.

## 2. Materials and Methods

### 2.1. Data Source

This longitudinal cohort study used data from the Taiwan Longitudinal Study on Aging (TLSA), which has been conducted by the health promotion ministration since 1989. TLSA involves adults aged above 60 years residing in nonaboriginal townships of Taiwan. The respondents were followed every 3 to 4 years (1989, 1993, 1996, 1999, 2003, 2007, and 2011). Two fresh samples were added in 1996 and 2003 to maintain the representativeness of the younger age cohort and extend that of the cohort aged 50 years or more. This trend analysis obtained data from wave 3 and examined the multimorbidity patterns in 1996, 1999, 2003, 2007, and 2011 separately. Initially, 5130 individuals aged above 50 years were involved, and in 2011, 2420 individuals were included in the analysis.

The mortality rate was verified in 2012 using the Death Registration from the Ministry of the Interior in Taiwan.

### 2.2. Study Variables

This study assessed 12 diseases, including hypertension, diabetes mellitus, coronary artery disease, stroke, cancer, lung disease, arthritis or rheumatic disease, hepatobiliary disease, renal disease (including stone), gout, hip fracture, and cataracts. Participants were asked the following question: “Have you ever had the disease…?” If the answer was “No” or “I don’t know”, they would be categorized as the disease-free group. Other variables were age, sex, income level, social participation, self-rated health, health behaviors (smoking, drinking, betelnut chewing, and exercise habit), admission experience in the past 12 months, disability, and depression.

The level of income was determined by asking “Are you satisfied with your income?” The answer could be good (very satisfied/satisfied), fair, or poor (unsatisfied/very unsatisfied). Individuals who had either paid, voluntarily worked, or participated in community activities were considered as having social participation. Moreover, individuals were divided into three groups according to self-rated health: good (very good/good), fair, and poor (poor/very poor). Exercise habits were divided into no exercise, ≤2 times, 3–5 times, and ≥6 times per week.

Their activities of daily living were also assessed by asking if they can do the following tasks: bathing, taking off and putting on clothes, eating meal, getting up from bed, standing and sitting on a chair, walking indoor, and going to the toilet. If they could not do any one of these tasks, they were considered disabled. In addition, depression was evaluated using the 10-item questionnaire of the Center for Epidemiologic Studies Depression Scale (CES-D). Each question was scored between 0 and 3, and the last two questions were reverse questions. A score above 10 points indicated depression [14].

### 2.3. Statistical Analysis

Disease patterns were estimated by LCA. We chose the most appropriate model groups with lower Bayesian Information Criterion values and descriptively analyzed the demographic and clinical characteristics of each group. Continuous and categorical variables were assessed using the analysis of variance and Chi-square test, respectively. The relationship between disease patterns and mortality was examined by univariate and multivariate logistic regression. In the multivariate analysis, we classified all the covariates into sociodemographic factors (age, sex, income satisfaction, and social participation), health behavior factors (smoking, drinking, and exercise habits), and health status factors (self-rated health, admission experience, disability status, and depression status). Each time we added one group, we adjusted the covariates to observe the effect of different dimensions.

The LCA was performed in PROC LCA 1.3.2, which is developed for SAS version 9.4 for Windows by the Methodology Center at Penn State. All the data were analyzed using the SAS 9.4 (SAS Institute, Cary, NC, USA). A *p*-value of less than 0.05 was considered statistically significant.

## 3. Results

**Figure 1:**
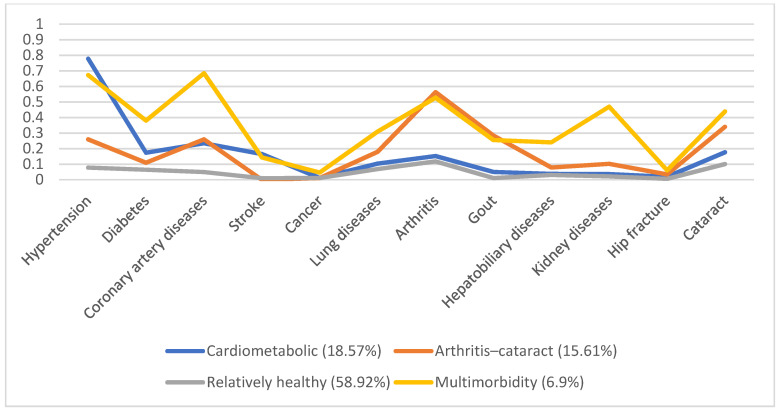
Multimorbidity patterns in 1996.

**Figure 2:**
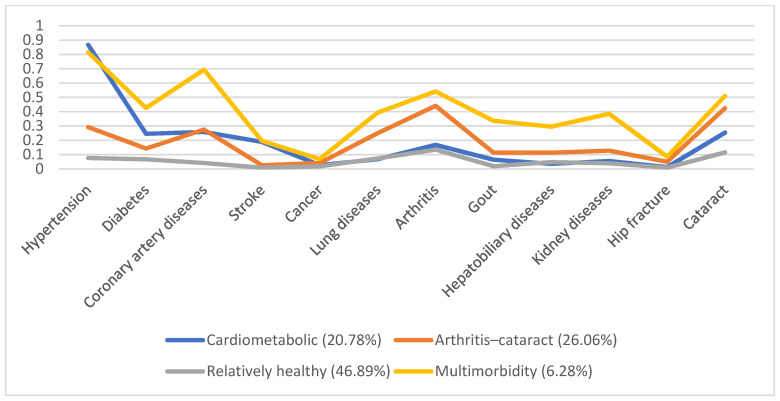
Multimorbidity patterns in 1999.

**Figure 3:**
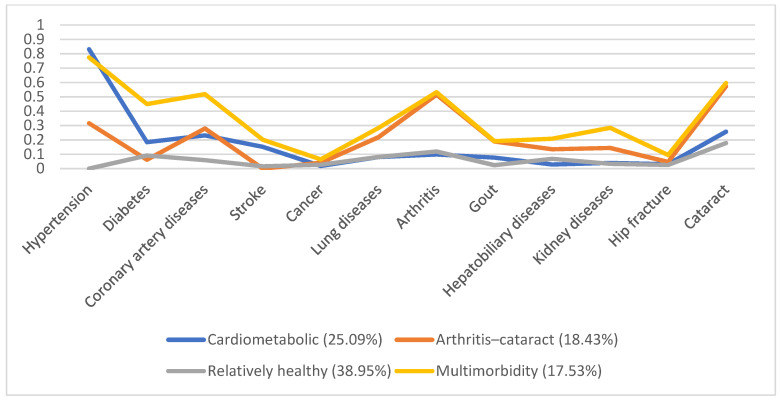
Multimorbidity patterns in 2003.

**Figure 4:**
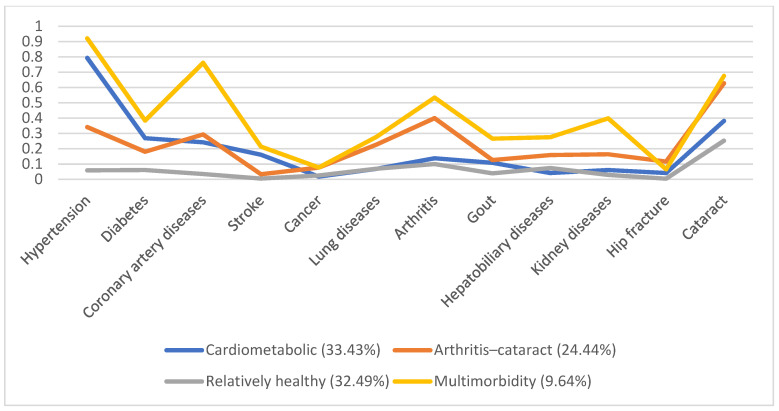
Multimorbidity patterns in 2007.

**Figure 5:**
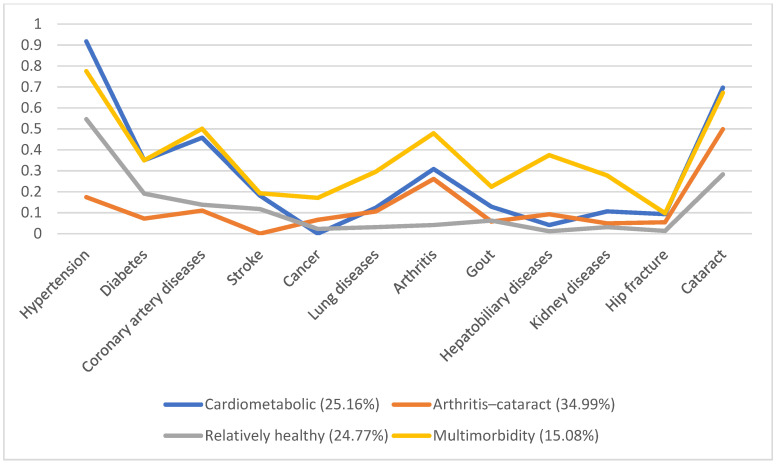
Multimorbidity patterns in 2011.

In 1996, 5130 individuals were involved, with male predominance (53.8%) and a mean age of 66.7. Additionally, we identified four disease patterns, namely, the cardiometabolic, arthritis–cataract, relatively healthy, and multimorbidity groups (18.57%, 15.61%, 58.92%, and 6.9%, respectively). These disease patterns remained similar in the following years (Figure 1, Figure 2, Figure 3, Figure 4 and Figure 5). However, eventually, the participants acquired more disease problems, causing the proportion of the multimorbidity group to rise from 6.9% to 15.08% and that of the relatively health group to decrease from 58.92% to 24.77%.

The baseline demographic characteristics showed higher rates of poor income satisfaction, self-rated health, admission experience, disability, and depression in the multimorbidity group than in the other groups (Table 1).

**Figure 6:**
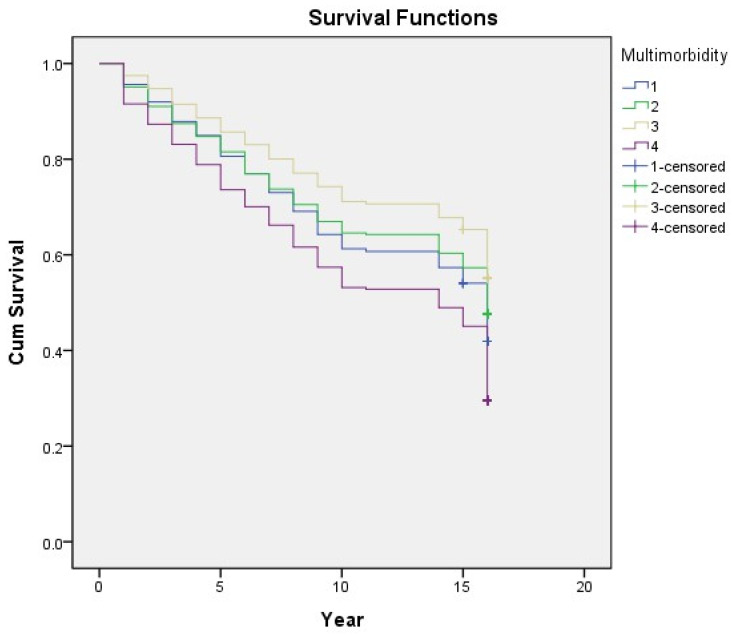
Association between each multimorbidity group in 1996 and long-term mortality in older adults. 1 (blue line): cardiometabolic group; 2 (green line): arthritis–cataract group; 3 (yellow line): relatively healthy group; 4 (purple line): multimorbidity group. Cum survival: cumulative survival.

In the univariate logistic regression analysis, all the variables, except for betelnut chewing, were significantly associated with mortality (Table 2). In the unadjusted model (Model 1), the multimorbidity group showed a higher risk for mortality, followed by the cardiometabolic and arthritis–cataract groups. Figure 6 shows the survival analysis of the Kaplan–Meier curve under the Cox regression model. After adjusting the socioeconomic factors (Model 2), the relationship between mortality risk and the arthritis–cataract group became insignificant. Model 3 (added health behavior confounders) demonstrated that the mortality risk was high in individuals with a smoking habit and that drinking showed a protective effect; the result remained the same in Model 4 (added health status factors). Eventually, after adjusting all the confounders, the cardiometabolic group showed the highest risk for mortality (odd ratio: 1.237, 95% confidence interval: 1.040–1.472); other significant risk factors were advanced age, male sex, smoking, poor self-rated health, admission in the past year, disability, and depression (Table 3).

Subgroup analysis of age among different multimorbidity patterns in relation to mortality was also done. There was a significant association between the cardiometabolic group and multimorbidity group and mortality among participants less than 65 years old. Gender has no effect on mortality in different multimorbidity patterns according to the subgroup analysis (Supplementary Table S1).

## 4. Discussion

In this population-based longitudinal study with LCA, we determined four disease patterns, namely, the cardiometabolic, arthritis–cataract, relative healthy, and multimorbidity groups. Comparing our findings with those of other countries is difficult because the population compositions and socioeconomic status are very different. Nonetheless, our findings and those from other countries have some similarities. For instance, the relatively healthy group is the majority, and the percentage is approximately 50–70% [12,13,15,16]. In our study, the relatively healthy group accounted for 58.92% of the whole study population.

Hypertension, diabetes, coronary diseases, and stroke, which constitute the cardiometabolic group, coexist in many studies [12,13,16]. The World Health Organization and the American Society of Endocrinology already recognize cardiometabolic syndrome as a disease entity [17] with similar risk factors and pathophysiology, such as visceral obesity, chronic inflammation, insulin resistance, dyslipidemia, and hypertension. In our study, the cardiometabolic group comprised almost 20% of older people initially and had a higher risk for mortality in the multivariate regression analysis. Developing an effective screening strategy to identify the population at risk and introducing appropriate treatment and lifestyle interventions are essential among older people [18]. Furthermore, our results implicated that healthcare professionals should be more careful when treating patients with comorbid hypertension, diabetes, coronary diseases, and stroke, especially those less than 65 years old. Case management and telemonitoring should be applied to this specific group of patients to lower the risk in daily life [19,20].

Our study also identified the arthritis–cataract group, which is not frequently seen in other studies. Only few studies have investigated the relationship between eye diseases and arthritis. One study using data from the Irish Longitudinal Study on Aging found that eye diseases increased the risk of developing arthritis, whereby cataracts were the most significant [21]. In other studies, ocular comorbidities were associated with many types of arthritis, including juvenile idiopathic arthritis and psoriatic arthritis [22,23]. Although the causal relationship is vague, some common pathological mechanisms are involved. For example, the inflammatory process may both affect the joints and eyes [24,25]. A unifying role of the oxidative stress between cataracts and rheumatoid arthritis has also been suggested [26]. The body’s immune system attacking self-antigens also plays a crucial role in arthritis and ocular comorbidities [27]. Corticosteroid could also be a linkage between these two comorbidities. A meta-analysis found that there is a possible association between glucocorticoid use and the development of cataract in patients with rheumatoid arthritis [28]. In a recent study, a relationship has been found between cataracts and juvenile arthritis mediated by topical corticosteroid use, suggesting that medication is one of the reasons for these comorbidities [29]. Additionally, life quality, negative self-perceptions regarding aging, mobility, memory impairments, and sleep quality mediated the relationship between cataracts and arthritis according to a previous study [21]. Further study is warranted to explore the mechanism of these two comorbidities. Although the arthritis–cataract group did not have a higher risk of mortality, many studies investigating both of these diseases reported a poorer quality of life [30,31,32,33,34]. To achieve a better quality of life, older people with these problems must receive early intervention and proper treatment.

Our stepwise multivariate analysis revealed that advanced age, male sex, smoking, poor self-rated health, admission in the past year, disability, and depression are risk factors of mortality. Interestingly, drinking was a protective factor in our analysis. Previous reports regarding the relationship between alcohol consumption and mortality were inconsistent [35,36,37]. Further study is required to evaluate the details of drinking habits and the types and amount of alcohol.

This study is the first to use LCA to evaluate disease patterns in Taiwan. Moreover, it included a large nationwide, representative, and randomly selected population with extremely high response rates. Hence, the results are reliable, thereby applicable for risk stratification by policymakers and the development of effective health interventions.

However, this study has some limitations. First, all the variables obtained were self-reported. Although some questions, such as “Is your disease being diagnosed by doctors or treated with medications?” were added to improve the accuracy; no medical records, blood tests, or images were utilized to confirm the diagnosis. Recall bias may also exist. Second, the relationship between each disease pattern and mortality was surveyed over a long period. The time-varying effect was not considered in the logistic regression model. Further statistical methods concerning the time effect might be used in future studies.

## 5. Conclusions

This nationwide study identified four disease patterns in older people: the cardiometabolic, arthritis–cataract, relatively healthy, and multimorbidity groups. The cardiometabolic group showed the highest risk for mortality. Thus, improving the prevention strategy toward cardiometabolic diseases with proper intervention should be emphasized in the future.

## Figures and Tables

**Table 1:** Demographic and clinical characteristics of the participants in 1996.

				Multimorbidity Patterns			
	Characteristics	Total	Cardiometabolic	Arthritis–Cataract	Relatively Healthy	Multimorbidity	*p* Value
		*n* = 5130	*n* = 1111	*n* = 594	*n* = 3138	*n* = 287	
Age		66.7 (9.37)	68.12 (8.84)	68.9 (8.76)	65.51 (9.58)	69.73 (7.9)	<0.0001
Sex							<0.0001
	Male	2760 (53.8%)	577 (51.94%)	258 (43.43%)	1794 (57.17%)	131 (45.64%)	
	Female	2370 (46.2%)	534 (48.06%)	336 (56.57%)	1344 (42.83%)	156 (54.36%)	
Income satisfaction							<0.0001
	Poor	829 (17.79%)	166 (16.73%)	106 (18.76%)	478 (16.35%)	79 (30.38%)	
	Fair	2126 (44.3%)	432 (43.55%)	271 (47.96%)	1310 (44.82%)	93 (35.77%)	
	Good	1805 (38.08%)	394 (39.72%)	188 (33.27%)	1135 (38.83%)	88 (33.85%)	
Social participation							<0.0001
	Yes	2146 (41.83%)	509 (45.81%)	259 (43.6%)	1234 (39.32%)	144 (50.17%)	
	No	2984 (58.17%)	602 (54.19%)	335 (56.4%)	1904 (60.68%)	143 (49.83%)	
Self-rated health							<0.0001
	Poor	1401 (29.40%)	359 (35.83%)	261 (46.03%)	594 (20.24%)	187 (71.37%)	
	Fair	1613 (33.84%)	341 (34.03%)	205 (36.16%)	1008 (30.34%)	59 (22.52%)	
	Good	1752 (36.76%)	302 (30.14%)	101 (17.81%)	1333 (45.42%)	16 (6.11%)	
Smoking							<0.0001
	Yes	1380 (26.9%)	224 (20.14%)	123 (20.71%)	971 (30.94%)	62 (21.6%)	
	No	3750 (73.1%)	887 (79.84%)	471 (79.29%)	2167 (69.06%)	225 (78.4%)	
Alcohol							<0.0001
	Yes	1083 (21.12%)	195 (17.55%)	98 (16.5%)	764 (24.35%)	26 (9.06%)	
	No	4046 (78.88%)	916 (82.45%)	496 (83.5%)	2373 (75.65%)	261 (90.94%)	
Betelnut							0.0212
	Yes	346 (6.75%)	65 (5.85%)	39 (6.57%)	233 (7.43%)	9 (3.14%)	
	No	4783 (93.25%)	1046 (94.15%)	555 (93.43%)	2904 (92.57%)	278 (96.86%)	
Admission in past one year							<0.0001
	Yes	909 (17.72%)	262 (23.58%)	129 (21.72%)	400 (12.75%)	118 (41.11%)	
	No	4221 (82.28%)	849 (76.42%)	465 (78.28%)	2738 (87.25%)	169 (58.89%)	
Exercise							0.0077
	No	2506 (48.87%)	488 (43.92%)	306 (51.52%)	1556 (49.62%)	156 (54.36%)	
	≤2 times/week	275 (5.36%)	54 (4.86%)	29 (4.88%)	174 (5.52%)	18 (6.27%)	
	3–5 times/week	395 (7.7%)	88 (7.92%)	43 (7.24%)	242 (7.72%)	22 (7.67%)	
	≥6 times/week	1952 (38.07%)	481 (43.29%)	216 (36.36%)	1164 (37.12%)	91 (31.71%)	
Disability							<0.0001
	Yes	671 (13.1%)	200 (18.03%)	111 (18.69%)	271 (8.64%)	89 (31.12%)	
	No	4453 (86.9%)	909 (81.97%)	483 (81.31%)	2864 (91.36%)	197 (68.88%)	
Depression							<0.0001
	Yes	1062 (22.42%)	228 (22.85%)	198 (35.17%)	508 (17.42%)	128 (49.61%)	
	No	3674 (77.58%)	770 (77.15%)	365 (64.83%)	2409 (82.58%)	130 (50.39%)	

The data in tables are the number (%) for categorical variables and the mean (SD) for continuous variables. OR, odds ratio; CI, confidence interval.

**Table 2:** Univariate logistic regression analysis of demographic and clinical characteristics predicting mortality.

		Mortality		
		OR	95% CI	*p* Value
Age		1.141 *	1.132–1.150	<0.0001
Sex				
	Male	1.551 *	1.389–1.732	<0.0001
	Female	Ref		
Income satisfaction				
	Poor	1.200 *	1.018–1.415	0.0299
	Fair	0.986	0.869–1.119	0.8271
	Good	Ref		
Social participation				
	Yes	Ref		
	No	1.716 *	1.534–1.919	<0.0001
Self-rated health				
	Poor	2.533 *	2.193–2.926	<0.0001
	Fair	1.356 *	1.182–1.556	<0.0001
	Good	Ref		
Smoking				
	Yes	1.44 *	1.272–1.630	<0.0001
	No	Ref		
Drinking				
	Yes	0.739	0.646–0.846	<0.0001
	No	Ref		
Betelnut				
	Yes	0.891	0.716–1.109	0.3013
	No	Ref		
Admission				
	Yes	2.719 *	2.333–3.169	<0.0001
	No	Ref		
Exercise habits				
	No	1.329 *	1.073–1.646	0.0092
	≤2 times/week	0.809	0.591–1.181	0.1865
	3–5 times/week	Ref		
	≥6 times/week	1.336 *	1.074–1.661	0.0092
Disability				
	Yes	5.669 *	4.618–6.958	<0.0001
	No	Ref		
Depression				
	Yes	2.203 *	1.760–2.325	<0.0001
	No	Ref		

OR, odds ratio; CI, confidence interval; *: *p* value < 0.05.

**Table 3:** Multivariate logistic regression analysis of demographic and clinical characteristics predicting mortality. Model 1: Disease patterns only; Model 2: Adjusted with socioeconomic factors; Model 3: Adjusted with socioeconomic factors and health behaviors; Model 4: Adjusted with socioeconomic factors, health behaviors, and physical conditions.

		Model 1			Model 2			Model 3			Model 4		
		OR	95% CI	*p* Value	OR	95% CI	*p* Value	OR	95% CI	*p* Value	OR	95% CI	*p* Value
Disease patterns													
	Cardio-metabolic	1.703 *	1.484–1.956	<0.0001	1.354 *	1.147–1.599	0.0003	1.424 *	1.203–1.684	<0.0001	1.237 *	1.040–1.472	0.0162
	Arthritis–cataract	1.379 *	1.157–1.644	<0.0001	1.023	0.831–1.260	0.8292	1.04	0.844–1.282	0.7145	0.831	0.667–1.034	0.0968
	Relatively healthy	Ref			Ref			Ref			Ref		
	Multimorbidity	2.924 *	2.251–3.797	<0.0001	2.028 *	1.504–2.736	<0.0001	2.008 *	1.486–2.713	<0.0001	1.353	0.982–1.863	0.0646
Age					1.136 *	1.126–1.146	<0.0001	1.14 *	1.129–1.150	<0.0001	1.133 *	1.122–1.143	<0.0001
Sex							<0.0001			<0.0001			<0.0001
	Male				1.907 *	1.659–2.192		1.689 *	1.437–1.985		1.718 *	1.454–2.028	
	Female				Ref			Ref			Ref		
Income satisfaction													
	Poor				1.54 *	1.269–1.870	<0.0001	1.434 *	1.177–1.747	0.0003	1.115	0.901–1380	0.3163
	Fair				1.137	0.981–1.316	0.0878	1.085	0.935–1.259	0.2826	1.013	0.869–1.181	0.8704
	Good				Ref			Ref			Ref		
Social participation							0.0023			0.0058			0.1369
	Yes				Ref			Ref			Ref		
	No				1.247 *	1.082–1.437		1.224 *	1.060–1.413		1.119	0.965–1.297	
Smoking										<0.0001			<0.0001
	Yes							1.708 *	1.432–2.037		1.756 *	1.468–2.1	
	No							Ref			Ref		
Drinking										0.0003			0.0197
	Yes							0.724 *	0.606–0.864		0.807 *	0.673–0.966	
	No							Ref			Ref		
Exercise habits													
	No							1.222	0.945–1.582	0.1267	1.172	0.901–1.525	0.2358
	≤2 times/week							0.888	0.605–1.302	0.5416	0.872	0.591–1.287	0.4903
	3–5 times/week							Ref			Ref		
	≥6 times/week							0.97	0.748–1.259	0.8193	1.06	0.814–1.382	0.6649
Self-rated health													
	Poor										1.587 *	1.301–1.935	<0.0001
	Fair										1.119	0.947–1.323	0.1853
	Good										Ref		
Admission													<0.0001
	Yes										1.84 *	1.516–2.232	
	No										Ref		
Disability													<0.0001
	Yes										1.799 *	1.390–2.2328	
	No										Ref		
Depression													0.0178
	Yes										1.249 *	1.039–1.501	
	No										No		

OR, odds ratio; CI, confidence interval; *: *p* value < 0.05.

## Data Availability

The datasets used and analyzed during the current study are not publicly available, but are available from the corresponding author on reasonable request with the permission of the Ministry of Health and Welfare, Taiwan.

## Supplementary Materials

The following supporting information can be downloaded at: https://www.mdpi.com/article/10.3390/ijerph19063317/s1, Table S1. Subgroup analysis of age among different multimorbidity patterns in relation to mortality.
