# Supplementary material for: Trends of Multimorbidity Patterns over 16 Years in Older Taiwanese People and Their Relationship to Mortality
Source: Int J Environ Res Public Health. 2022 Mar 11;19(6):3317. doi: 10.3390/ijerph19063317 (PMC8950835; doi:10.3390/ijerph19063317)
Supplement: Supplementary file 1 [file ijerph-19-03317-s001.zip › ijerph-1590279-supplementary.pdf]

Table S1. Subgroup analysis of age among different multimorbidity patterns in relation to mortality.

		Multimorbidity Patterns			
		**Cardiometabolic**	**Arthritis–Cataract**	**Relatively Healthy**	**Multimorbidity**
Age					
	≥65(3037)	1.161 (0.944–1.427)	0.876 (0.683–1.125)	Ref	1.114 (0.769–1.614)
	<65(2094)	1.378 (1.039–1.829)	0.821 (0.540–1.247)	Ref	2.220 (1.273–3.873)

Confounding factors: age, income level, social participation, self-rated health, admission in the past year, disability, depression, smoking. The data in the tables are odds ratios with confidence interval (CI).
